# Some Recent Advances in Physiology and Pathology
1Practitioner, May, 1905.


**Published:** 1905-05-20

**Authors:** 


### SOME RECENT ADVANCES IN PHYSIOLOGY AND PATHOLOGY.[1]

(*Concluded from page* 117.)

*The Thyroid Gland.*—Severe typical tetany may be induced by operative suppression of thyroid and parathyroid tissues, and is at the same time not infrequent as a closing incident of exophthalmic goitre. Tetany has also been from time to time observed as a passing condition during the pregnancies of certain sufferers from goitre, both exophthalmic and simple. In view of these facts some have attempted thyroid treatment for tetany occurring in pregnancy, and apparently with success. It seems to be fairly clear that the symptoms of exophthalmic goitre are not due to the mere escape into the tissues of an excessive thyroid secretion, for experimental injection of a watery extract of thyroid gland habitually causes a fall of blood-pressure, whereas a diminution of blood-pressure in Graves' disease appears to be the exception rather than the rule. There are certain cases of thyroid disorder which are neither myxœdema nor exophthalmic goitre, but partake of the characteristics of both these diseases, a group denoted by French physicians as cases of "dysthyroidism." A case of the kind is reported by V. Schrötter in which there was marked pigmentation followed by a myxœdematous swelling of the legs and arms, that is to say a swelling which did not pit on pressure. The swelling was subject to great and rapid fluctuations, compared by von Schrötter to the *crises d'amaigrissement* described by Huchard in certain cases of exophthalmic goitre. The case in point was much benefited by thyroid administration, in the course of which the swelling was reduced, the thyroid enlargement lessened and the tachycardia abated. In very severe cases of exophthalmic goitre, where corneal ulcers threaten, or other complications dangerous to life, removal of a portion of the cervical sympathetic has been practised with a certain degree of success; but its risks are grave.

*The Supra-renal Glands.*—The medullary portion of the supra-renals is in direct developmental relationship with the sympathetic nervous system. In many cases of Addison's disease a tuberculous process is found to involve the medullary portions of these bodies, but not in all. Furthermore, experimental ablation of these bodies has failed to reproduce the clinical picture of Addison's disease. These contradictions are explained by a hypothesis of Wiesel. He finds that there are three main elements in the sympathetic system—ganglion cells, nerve-fibres and chromophile cells, so called because they give a brown colour when treated with chromic acid. These chromophile cells occur in all parts of the system, and are identical with those found in the medullary portions of the supra-renal bodies. Wiesel examined microscopically the tissues of five cases of Addison's disease, and found that the chromophile cells had entirely disappeared, while the supra-renals, with the exception of the medullary parts, were intact. He concludes that Addison's disease is a primary disease of the chromophilic system of cells, with secondary involvement of the sympathetic system, and possibly of the cortical portions of the supra-renal glands.

[1] Practitioner, May, 1905.

